# A multiplex platform for digital measurement of circular DNA reaction products

**DOI:** 10.1093/nar/gkaa419

**Published:** 2020-05-29

**Authors:** Johan Björkesten, Sourabh Patil, Claudia Fredolini, Peter Lönn, Ulf Landegren

**Affiliations:** From the department of Immunology, Genetics and Pathology, Science for Life Laboratory, Uppsala University, Uppsala, 751 08, Sweden; From the department of Immunology, Genetics and Pathology, Science for Life Laboratory, Uppsala University, Uppsala, 751 08, Sweden; From the department of Immunology, Genetics and Pathology, Science for Life Laboratory, Uppsala University, Uppsala, 751 08, Sweden; From the department of Immunology, Genetics and Pathology, Science for Life Laboratory, Uppsala University, Uppsala, 751 08, Sweden; From the department of Immunology, Genetics and Pathology, Science for Life Laboratory, Uppsala University, Uppsala, 751 08, Sweden

## Abstract

Digital PCR provides high sensitivity and unprecedented accuracy in DNA quantification, but current approaches require dedicated instrumentation and have limited opportunities for multiplexing. Here, we present an isothermal platform for digital enumeration of DNA reaction products in multiplex via standard fluorescence microscopy. Circular DNA strands, which may result from a wide range of molecular detection reactions, are captured on streptavidin-coated surfaces via hybridized biotinylated primers, followed by rolling circle amplification (RCA). The addition of 15% polyethylene glycol 4000 during RCA resulted in uniform, easily recorded reaction products. Immobilized DNA circles were visualized as RCA products with 100% efficiency, as determined by droplet digital PCR. We confirmed previous reports about the influence on RCA by sequence composition and size of RCA templates, and we developed an efficient one-step restaining procedure for sequential multiplexing using toehold-triggered DNA strand displacement. Finally, we exemplify applications of this digital readout platform by demonstrating more than three orders of magnitude improved sensitivity by digital measurement of prostate specific antigen (PSA) (detection threshold ∼100 pg/l), compared to a commercial enzyme-linked immunosorbent assay (ELISA) with analogue readout (detection threshold ∼500 ng/l), using the same antibody pair.

## INTRODUCTION

There is an increasing need to digitally enumerate individual biomolecules and molecular events, as a sensitive and accurate means to quantify features in biological samples. Also, the growing understanding of disease complexity creates a need for highly multiplex measurements of many biomarkers of one or more molecular classes. Digital PCR (dPCR) offers unprecedented accuracy for enumerating individual DNA molecules in a sample (1), and the technique is increasingly replacing analogue measurements of the fractional cycle when a given level of fluorescence is exceeded in real-time PCR, or the still earlier estimation of amounts of endpoint PCR products by gel electrophoresis. However, dPCR suffers from limitations such as the requirement for dedicated instruments, high cost, low multiplexing and limited dynamic range.

Rolling circle amplification (RCA) is an efficient method to locally amplify individual circular DNA molecules to easily detectable levels. The technique serves to generate distinct μm-sized objects - rolling circle products (RCPs), each containing hundreds or more copies of the complement of each templating DNA circle (2). After specific staining with fluorophore-conjugated DNA oligonucleotides, individual RCPs can be visualized and distinguished from background as bright dots using fluorescence microscopy and computer-based image analysis, thereby permitting digital enumeration of products of reporter DNA circles as the output of molecular detection reactions that produce DNA circles.

Examples of detection reagents that generate circular reporter molecules include padlock probes for highly specific, multiplex DNA detection (3,4) and protein analyses undertaken either via immuno-RCA (5,6) or through *in situ* proximity ligation assays (isPLA) (7,8). These protein assays use detection reagents that are either equipped with preformed DNA circles for immunoRCA, or where DNA circles are formed upon proximal binding by pairs of antibodies in isPLA. The RCPs can be generated in solution and distributed onto a surface for enumeration (9,10), or the targets may be located on a support, such as for *in situ* analyses. In yet other assays circular DNA reaction products formed in solution may be captured on solid supports before RCA (11). The formation or capture of DNA circles on supports admits washes that ensure optimal RCA conditions by removing inhibitory sample components and/or excess reagents.

Polyethylene glycol (PEG) has previously been shown to influence the efficiency of RCA on magnetic beads (12). Also the size and sequence composition of the circular templates have been reported to affect the efficiency of RCA performed in solution (13,14). Here we have optimized RCA performed on streptavidin-coated microscope slides. We evaluated aspects such as the effect on RCA efficiency by the addition of PEG and by the circular template size and sequence composition in some detail. Our optimized reaction conditions were applied for digital detection of PSA in an assay that exceeded the sensitivity of detection of a commercial ELISA using the same antibody pair by three orders of magnitude.

## MATERIALS AND METHODS

### Workflow

**Figure 1.**
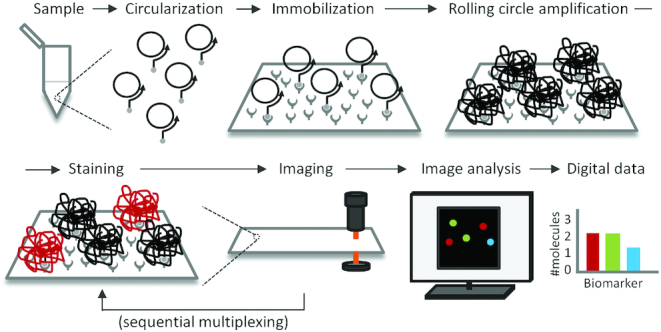
Workflow for digital detection of RCPs. First, a molecular assay results in the formation of DNA circles in response to the presence of target molecules (Circularization) and the DNA circles formed are captured, hybridized to biotinylated primers on a streptavidin coated microscope slide (Immobilization). RCA is then used to elongate the immobilized primers with the DNA circles as templates, resulting in long concatemeric DNA molecules that collapse into individual DNA bundles (rolling circle amplification – RCA). The next step (which can be performed simultaneously with the previous one) is to stain the RCPs with detection oligonucleotides conjugated to fluorophores (Staining), followed by recording the numbers of bright RCPs via fluorescence microscopy (Imaging). Finally, image analysis software is used to digitally enumerate the numbers of RCPs in each image and fluorescence channel (image analysis), followed by processing of the retrieved data using statistics software (data analysis). The staining and imaging steps can be repeated several times for sequential multiplexing. In this case, toehold-mediated DNA strand displacement was deployed to simultaneously de-stain previously imaged RCPs while staining new RCPs to be imaged.

The present DNA detection method consists of the following steps: generation of circular DNA strands, immobilization of the DNA circles on a planar support, and RCA followed by staining and imaging with data analysis (Figure 1). Staining and imaging can be repeated several times to achieve the desired degree of multiplexing. The de-staining is combined with staining of new sets of RCPs in a single reaction step.

### Circularization

DNA circularization is typically a consequence of detection reactions for target nucleic acids or proteins, but during development of this readout procedure, synthetic 5′ biotinylated DNA strands were used to template circularization of linear padlock probes. The template strands were designed to hybridize to at least 10 nucleotides at both the 3′ and 5′ ends of the padlock probes, to allow for efficient circularization. Sequences of all oligonucleotides used are documented in Table 1. Ligation was typically performed by mixing target molecules and biotinylated ligation templates at 100 nM each in a 100 μl reaction mix containing 1× T4 DNA ligase buffer, 1 mM ATP and 4 units T4 DNA ligase (all from Thermo Fisher Scientific, USA). The ligation mix was incubated at room temperature for 90 min to allow efficient circularization. To evaluate the ligation process via gel electrophoresis 8.5 μl of the circle ligation reaction was mixed with 0.5 μl each of exonuclease I (20 U/μl), exonuclease III (200 U/μl) and lambda exonuclease (10 U/μl) (all from Thermo Fisher Scientific, US) and incubated at 37°C for 60 min and 95°C for 10 min (Supplementary Figures S4 and S7) to remove any remaining linear oligonucleotides. Circularized target molecules were kept frozen at high concentration and diluted just before immobilization. No loss of the circular DNA stock material was observed upon many freeze-thaw cycles. Denaturing polyacrylamide gel electrophoresis was used to verify efficient circularization (Supplementary Figures S4 and S7). A Novex™ TBE Urea gel system (Thermo Fisher Scientific, USA) with 10% gels were used. To ensure denaturing conditions at an elevated temperature, the gel cassette was partly submerged into a 45°C water bath during electrophoresis. 10 μl sample was loaded per well (5 μl sample and 5 μl 2× TBE urea loading buffer), and the gel was run at 180 V for 30 min before staining with 1× SYBR Gold for 15 min and imaging with a Bio-Rad ChemiDoc XRS+ system (Bio-Rad Laboratories, USA).

**Table 1.** The oligonucleotides used can be grouped into three distinct categories dependent on detection specificity (systems A, B and C). The coloring used in the sequences represents reverse complementary parts. The letter ‘N’ in the sequences represents a random nucleotide (A, T, C or G) and hence these oligonucleotides are pools of distinct sequences

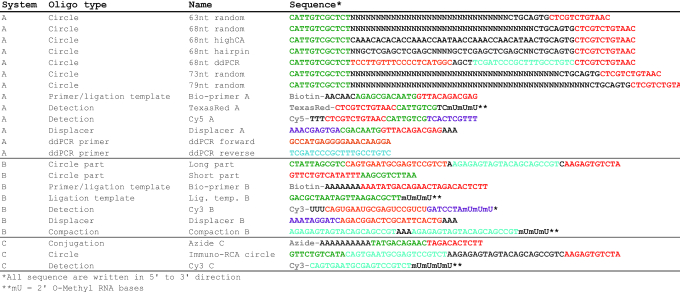

### Immobilization

DNA circles were immobilized on streptavidin-coated surfaces via hybridized, biotinylated oligonucleotides that also served as primers for RCA. Sixteen-well adhesive FlexWell™ chambers (Grace Bio-labs, USA) were attached on top of streptavidin-coated TRIDIA BA microscope slides (SurModics IVD Inc., USA) and 40 μl/well of DNA circles and primers were added in a Binding & Wash buffer consisting of 5 mM Tris (pH 7.5), 0.5 mM EDTA, 0.05% (v/v) Tween20 and 1 M NaCl. The chambers were sealed with adhesive PCR film and placed in humidity chambers (plastic boxes with deionized water and a rack to support the slide) in a 37°C incubator for 2 h. After immobilization the chambers were quickly washed twice with 100 μl/well Binding & Wash buffer, before proceeding with RCA. In an alternative approach, immobilizations by drying was investigated by adding 1 μl of 1 pM circular templates and primers in PBS or in PBS with 0.05% Tween20 (PBST) or in PBST supplemented with 15% PEG 4000 to a streptavidin-coated microscope slide, followed by incubation for a few minutes in a 45°C incubator. Reaction chambers were applied around the dried areas after complete drying. The efficiency of capture of DNA circles and detection of RCPs for circles dried in PBST supplemented with PEG, was calculated based on the theoretical number of applied circles, the diameter of the entire dried area, the size of one image, the average number of RCPs in one image, assuming that the RCPs were evenly distributed.

### RCA

RCA was performed by adding 40 μl/well reaction mixture containing Phi29 DNA polymerase reaction buffer (Monserate Biotechnology Group, USA), 0.25 mM dNTPs (Thermo Fisher Scientific), 8 units Phi29 DNA polymerase (Monserate Biotechnology Group, USA) and 15% PEG 4000 (Thermo Fisher Scientific) unless stated otherwise. The chambers were sealed with adhesive PCR film and placed in a humidity chamber in a 37°C incubator for 2 h. After immobilization, the chambers were quickly washed twice with 100 μl/well Binding & Wash buffer before proceeding with staining. Titrations of PEG have been analyzed in multiple experiments, consistently identifying 15% as an optimal concentration.

### Staining

Staining was performed by adding 40 μl/well staining solution containing Binding & Wash buffer, 10% Dextran Sulfate (50% Solution from Millipore, USA) and 100 nM detection oligonucleotide. The slides were incubated for 10 min in a 37°C incubator without sealing, before removal of the incubation chamber, washing twice for five min in Binding & Wash buffer in a cuvette on a shaker plate set to low speed, quick dehydration in an ethanol series and mounting with SlowFadeGold mounting medium (Thermo Fisher Scientific).

### Imaging

Imaging was performed with a Zeiss Imager Z2 microscope equipped with a Hamamatsu ORCA-flash4.0 LT digital camera using a 20× objective and manual focusing in live view. Three none overlapping images were acquired from each well and used to derive the data. Exposure times were in the range of 0.5–2 s for the different fluorescence channels. Imaging conditions were kept constant for each fluorescence channel in each experiment, and carefully adjusted not to include overexposed areas. No adjustments were made to the images before image analysis and data retrieval using the CellProfiler software (15). Brightness and contrast were adjusted for visualization purposes in the figures but kept constant within each experiment.

### Image analysis

RCP characterization and measurements of the different images were performed using the CellProfiler software (15). Small variations in the pipeline settings were done between the different experiments. A typical pipeline included five different modules to identify and measure RCPs in individual images, and export the retrieved data to a spreadsheet (Supplementary Figure S3). The settings were kept constant within each experiment with one exception; samples with no PEG present during RCA were analyzed using a lower threshold to identify RCPs (0.01 compared to 0.05) and without a method to draw dividing lines between clumped objects, compared to samples with PEG. This disparity was due to the major difference in intensity and shape of the RCPs in these samples. The exact settings used for each experiment were carefully adjusted by using the Test Mode to manually inspect the output of the software.

### Data analysis

**Figure 2.**
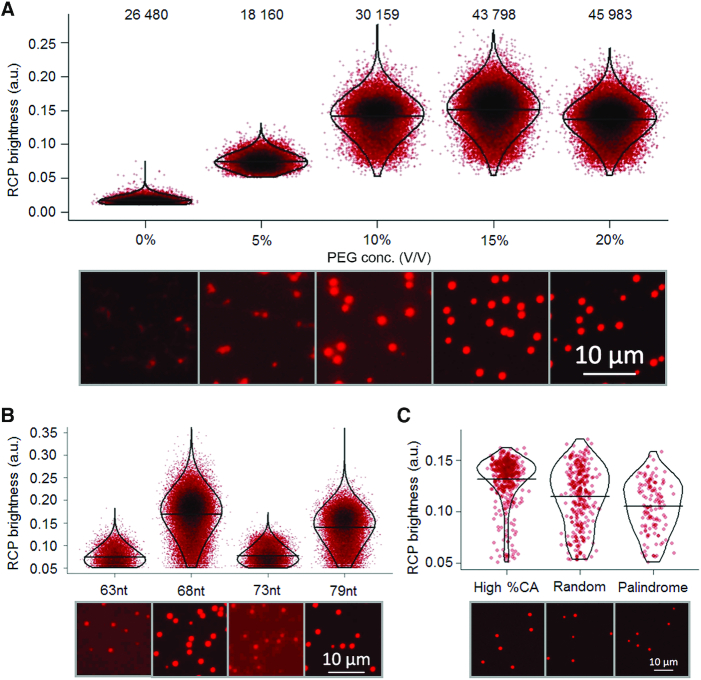
Conditions for efficient RCA were investigated by recording numbers of detected signals and the brightness of individual RCP. Each dot in the graphs represents the brightness of an individual RCP (calculated as the ratio between the intensity and area). For each investigated condition the dots in the graphs were distributed along the x-axis according to a normal distribution. Dots that are overlapping are shown in increasingly dark color. The distributions are overlaid with violin plots and the median brightness is indicated with horizontal lines. The efficiencies were also investigated via visual interpretation of microscope images with increased brightness and contrast. (**A**) RCA efficiencies were evaluated with increasing concentrations of PEG 4000 added. The numbers of RCPs recorded in the three images analyzed for each sample are displayed at the top. (**B**) RCPs resulting from circular templates of four different sizes (63, 68, 73 or 79 nucleotides) were compared. Circular DNA templates of 68 and 79 nucleotides produced brighter RCPs compared to 63 and 73 nucleotide circles. (**C**) 68 nucleotide long circular templates of different sequence composition were analyzed with respect to RCP brightness. Circular templates with high cytidine and adenosine content generated brighter RCPs that were more even in intensity, compared to a library with random sequences and a sequence with self-complementary palindromic elements.

**Figure 3.**
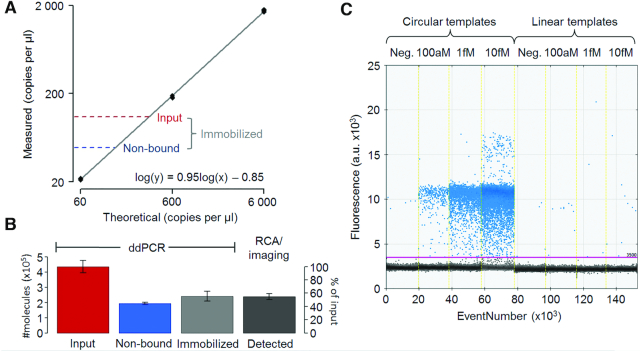
dPCR was used to evaluate efficiencies for capturing circular DNA molecules on a solid support and for locally amplifying them via RCA. (**A**) A 10-fold dilution series, analyzed in triplicate technical replicates, demonstrated excellent linearity (black line represents log-log linear regression). The numbers of circular templates added to the solid support (input) and the ones remaining in solution after immobilization (non-bound) were both within the linear range (red and blue dashed lines). (**B**) The difference between added molecules and ones remaining in solution was used as a measure of the numbers of circular templates immobilized on the solid support (slightly more than 50%). Enumeration via RCA and imaging demonstrated that all immobilized circular templates (light grey bar, defined by dPCR) gave rise to RCPs that could be recorded, as evidenced by the similar heights of the light and dark grey bars. (**C**) dPCR primers were designed to produce an amplicon over the ligation site so that any remaining uncircularized oligonucleotides would not be amplified. The dPCR specificity for circular templates was verified by analyzing a dilution series of ligated and non-ligated circular templates. Only circularized molecules generated efficient PCR amplification and hence positive droplets.

All data analyses were performed in R Studio, version 1.0.143. The distributions of RCP brightness for the different sample types in Figure 2 were calculated as the ratio between RCP Intensity and Area. The individual dots in the distribution plots were spread along the x-axis using a normal distribution function and overlaid with violin plots (vioplot package) and lines indicating the median brightness values. Input concentrations of DNA circles and measured output of amplicons from the dPCR analyses (molecules/μl) were plotted in log–log scatterplots together with a linear regression line based on the logarithmic values (Figure 3A). The numbers of molecules detected by imaging were calculated based on the ratio between the size of each image and the total area of the well, assuming that the signals were evenly distributed across each well (Figure 3B). The gray line in Figure 5A is a regression line based on the average number of RCPs per image for concentrations between 6 fM and 4 pM. In Figure 5C, the gray line represents the average number of RCPs per image for all samples except 1:10^5^.

### Oligonucleotides

All oligonucleotides were purchased from Integrated DNA Technologies (IDT (Table 1). The oligonucleotides were categorized into three different systems (A, B and C) based on their circularization and detection sequence specificities. Oligonucleotides from system A were used to evaluate the effects on RCA efficiency according to concentrations of PEG, circle length and circle sequence composition. Also dynamic range and immobilization and detection efficiencies (as determined via dPCR) were evaluated using system A. System B oligonucleotides were used to compare the effects on RCA by PEG and so-called compaction oligonucleotides, designed to condense RCPs (16). A combination of systems A and B was used to evaluate inhibition by saturation and for the sequential multiplexing demonstration. Oligonucleotides from system C were used to measure PSA via digital immuno-RCA.

### dPCR

The dPCR analysis was performed with a QX200™ AutoDG™ system and EvaGreen Supermix (Bio-Rad, USA) according to the manufacturer's instructions. The fluorescence intensity threshold for positive droplets was set manually, and the same threshold was used for all samples (Figure 3C). The dPCR runs were repeated on two occasions with closely similar results.

### Sequential multiplexing

RCPs arising from different DNA circles were sequentially identified by iteration of the staining and imaging steps to image successive sets of RCPs for multiplex detection (Figure 1). To remove detection oligonucleotides from previously imaged RCPs, specific displacement oligonucleotides were added along with fluorescence-labeled detection oligonucleotides for a new set of RCPs. The displacement oligonucleotides were fully complementary to previous sets of detection oligonucleotides, including a 10-nucleotide toehold region that was not complementary to the RCP. Hybridization to the toehold initiated a strand displacement process (17,18) which served to remove detection oligonucleotides from previously imaged RCPs. Thereby an excess of new detection oligonucleotides, as well as double stranded detection oligonucleotides from the earlier imaging cycle could both be removed by washes.

To demonstrate sequential multiplexing staining and displacement solutions were prepared with 100 nM detection oligonucleotide and 500 nM displacement oligonucleotide. This combined de-staining and staining solution was added directly to small areas on the slides defined with an ImmEDGE™ hydrophobic barrier pen (Vector Laboratories, UK). The slides were incubated for 8 min in a 37°C incubator and washed twice for 1 min in Binding & Wash buffer in a cuvette on a shaker plate at low speed. No dehydration of the slides was performed in the case of sequential multiplexing. Instead, a few drops of Binding & Wash buffer and coverslips were added to the slides before imaging. To remove the coverslips for subsequent staining cycles the slides were submerged in Binding & Wash buffer. With this protocol each one-step de-staining and new staining procedure was completed in about 10 min. The procedure has been repeated multiple times, varying conditions slightly during the optimization process.

### 96-well plate reader comparison

In order to compare digital and analog measurements of RCPs, we coated a microplate (Cell culture, 96 wells, PS half area, μ-clear, Black Advanced TC; Greiner Bio-One) with 200 nM recombinant streptavidin (Roche), diluted in coating buffer (bicarbonate/carbonate 100 mM pH 9.6) and incubated for 2 h at RT. The coating solution was then removed and the wells were washed with PBS. A dilution series between 100 pM and 100 aM of DNA circles hybridized to biotinylated primers was prepared in duplicate in Binding & Wash buffer. Immobilization, RCA and staining were performed as described above using volumes of 100 μl per well. Washes were performed with 200 μl of Binding & Wash buffer.

A Tecan Spark plate reader was used for analog readout, selecting a monochromatic acquisition (575–620 nm; band width 20 nm) and fluorescence bottom reading. Digital readout was performed on a Leica DMi8 inverted light microscope using a 20× dry objective (0.75.dry UV HC, PL APO CS2) and a filter set for TexasRed. Exposure times and imaging conditions were kept constant. Images were exported in TIFF format and analyzed with CellProfiler.

This entire experiment has been performed twice with closely similar results.

### Evaluation of binding capacity

To evaluate what total number of RCPs of any specific species could be recorded without inhibition in multiplexing situations 100 fM of one circular DNA strand was mixed with a distinct circular DNA strand at ratios; 1:0, 1:1, 1:10, 1:100, 1:1000, 1:10 000 and 1:100 000. The samples were immobilized on the solid support and analyzed as described above. Specific detection oligonucleotides conjugated to different fluorophores (Cy3 and Cy5) were used to stain the RCPs generated from the two different circular templates. This experiment was repeated once with the same outcome.

### Compaction oligonucleotides

We investigated how the shapes of RCPs were affected by addition of PEG 4000 compared to compaction oligonucleotides during RCA the same oligonucleotide sequences and protocol were used as described previously for successful compaction of RCPs (16), with and without addition of a final concentration of 15% PEG 4000 to the RCA mix, or with 15% PEG 4000 alone.

### ELISA measurement of PSA

A commercial ELISA kit for measurement of PSA (Fujirebio, CanAg PSA EIA, ref 340-10, IVD, CE_0197_) together with non-conjugated antibodies, were kindly provided by Dr Christian Fermér at Fujirebio. The kit consisted of two monoclonal anti-PSA antibodies; one conjugated to biotin for immobilizing the detection complex to a solid support (capture antibody) and the other conjugated to horseradish peroxidase (HRP) (detection antibody) to generate a detectable signal upon addition of 3,3′,5,5′-tetramethylbenzidine (TMB). The ELISA was performed according to the manufacturer's instructions. In brief, both capture and detection antibodies were incubated together with samples in streptavidin-coated strip wells (96-well format) for one hour at room temperature, followed by signal generation upon addition of a TMB solution and incubation for 30 min at room temperature. The signal was recorded as the absorbance at 420 nM using a Tecan Spark multimode microplate reader. Triplicate technical replicates were used for all evaluations and the absorbance values were log_10_-transformed for data visualization purpose. A nine-step calibration curve between 100 pg/l and 60 μg/l was prepared using the PSA calibrators included in the kit and analyzed together with a negative control. A six-step dilution series between 100 pg/l and 10 μg/l was prepared in pooled female plasma sample and analyzed together with non-spiked plasma. LOD values were calculated as concentrations yielding signals three standard deviations above the average background values. The sample amount in each well was 25 μl.

### Digital immuno-RCA measurement of PSA

The immuno-RCA analysis was performed using conditions as similar as possible to the ELISA measurement. The only difference in reagents was that the detection antibody was conjugated to a short DNA oligonucleotide with a free 3′ end (a detailed description is provided in the supplementary material), and further hybridized to a DNA circle. The DNA circle was generated by circularizing a linear DNA oligonucleotide using the antibody-conjugated oligonucleotide as template. This was performed using a five-fold molar excess of circularizable oligonucleotide over antibodies in a 20 μl ligation mix containing 1 μM conjugated antibody, 5 μM linear DNA oligonucleotide, 1xT4 ligation buffer, 1 mM ATP and 0.04 U/μl T4 DNA ligase (Thermo Fisher Scientific). The ligation mix was incubated for 60 min at room temperature. The antigen recognition was performed analogous to the ELISA kit with a 60 min one-step recognition and immobilization reaction at room temperature. The recognition reaction consisted of 30 μl PSA standard (the same dilution series as for the ELISA investigations described above) and 40 μl antibody mixture with 3.3 nM detection antibody and 6.7 nM capture antibody. The recognition incubation was performed in 16-well chambers on a streptavidin coated slide (used as described above). Also RCA, detection incubation and microscopy analysis were performed as described above with the only exception that dextran sulfate was excluded from the detection oligo staining mixture. Six images were captured from different location for each sample. Efficiency was calculated based on the stated amount of PSA in the calibrators added, the average number of signals in one image above LOD and the ratio between the area of one image and one well (0.96%) as

$$\begin{equation*}{\rm Eff}\left( \% \right) = \frac{{({N_{{\rm signals}}} - {\rm LOD})}}{{0.0096\ \times {N_{{\rm added}}}}}\ \times 100\end{equation*}$$

LOD values were calculated at three standard deviations above the average background counts. Digital immuno-RCA experiments were performed on multiple occasions during the optimization process, and the conclusions of each of these experiments were the same.

### Plasma samples

The male and female EDTA plasma samples used for PSA measurements were pools from several voluntary blood donors obtained from the blood central at Uppsala university hospital. The samples are fully anonymized and no personal or clinical data is available. No explicit ethical permit or specific consent was needed for these anonymized and pooled samples, according to Swedish law (2003:460) for Ethical Review of Research Involving Humans.

## RESULTS

A simplified model experiment, based on immobilization of DNA circles on streptavidin-coated microscope slides, was used to evaluate different properties associated with generation and digital recording of RCPs on solid support (Figure 1). We also evaluated a more realistic application of ultra-sensitive detection of PSA via a digital immuno-RCA assay and compared this to standard ELISA with analog readout.

### RCA efficiency

#### Effects by PEG and circular template size and sequence on RCA

We observed that the addition of PEG 4000 to a final concentration of 10–20% in the RCA reactions was helpful to produce RCPs in greater numbers that also were more similar in size and of generally higher intensity (Figure 2A and Supplementary Figure S1A). Without PEG the RCPs are quite faint, non-uniform and randomly extended on the surface, with several local spots of higher intensity, precluding accurate enumeration. The highest average intensity and uniformity of RCPs was consistently achieved when 15% PEG was used. Analysis using the CellProfiler software confirmed that the presence of 15% PEG resulted in the most rounded and homogenous population of RCPs, simplifying identification and digital recording (Supplementary Figure S2). This concentration was used for all subsequent experiments unless otherwise stated.

Joffroy and coworkers recently reported that the efficiency of RCA depends on the size of the DNA circle templating the reaction. The efficiency was shown to vary in a periodic fashion with a maximum every 10.5 nucleotide, a number that corresponds to the length of one turn of the DNA double helix (13). We were able to confirm these results, as our results clearly demonstrate that circular templates of 68 and 79 nucleotides (previously described maxima) generated considerably brighter RCPs compared to ones arising from circular DNA templates of 63 and 73 nucleotides (previously described minima) (Figure 2B and Supplementary Figure S1B). The brightest RCPs were achieved using a 68 nucleotide circular template and hence this template size was used in the following experiments, unless otherwise stated.

RCA efficiency has also been shown to depend on the sequence composition of the DNA circles, with high cytidine and adenosine content increasing efficiency of solution-phase RCA (14). We could confirm this claim also for RCA performed on planar solid supports, by demonstrating a higher average brightness for circular templates rich in cytidine and adenosine compared to a pool of oligonucleotides with segments of random sequence or a specific sequence that includes palindromic, self-complementary elements (Figure 2C and Supplementary Figure S1C). The palindromic elements evaluated are the ones used to generate compacted RCPs in solution as sequencing templates by the company Complete Genomics (San Jose, US) (19).

#### Immobilization and detection efficiencies

A dPCR assay was used to establish what fraction of circularized target molecules that could be captured on the streptavidin-coated surface, while digital enumeration of RCPs by microscopy was used to evaluate what fraction of the captured circular DNA strands that could form RCPs and thus be detected (Figure 3). A 10-fold dilution series of circular DNA templates was analyzed in triplicate by dPCR with very low variation and high linearity (Figure 3A). Both the measured input sample DNA molecules applied to the solid support and the DNA molecules that failed to attach to the support were in the linear dynamic range. We consistently observed that under the conditions used slightly more than 50% of the input molecules were attached to the solid support (based on measured input versus non-bound molecules), and all captured DNA circles were detected via RCA, imaging and counting of RCPs (Figure 3B). We confirmed that the dPCR assay only recognized circularized and not linear DNA templates, as demonstrated by comparing to linear templates added at different concentrations (Figure 3C). The oligonucleotides used were 68nt dPCR oligonucleotide (to be circularized), Bio-primer A and forward and reverse dPCR primers (Table 1).

We explored the possibility to speed-up immobilization of circular templates to the solid support by drying the sample on the streptavidin-coated surface. PBST with 15% PEG gave better results than when PBST or PBS was used without PEG, by producing a dried sample area with an even distribution of signals (Supplementary Figure S6). A prominent ‘coffee stain’ effect (enrichment of signals along the periphery) was seen in samples without PEG, in accordance with a previous report (20). The efficiency of immobilization and detection was relatively low, however, at around 1% of the theoretical maximum, and therefore considerably less efficient than incubation for two hrs without drying.

#### Effects by compaction oligonucleotides on RCPs

Compaction oligonucleotides are oligonucleotides that consist of two identical parts complementary to motifs in the repeated sequences that make up an RCP (16). The purpose of using compaction oligonucleotides is to bring distant regions of RCPs together and hence prevent spreading of RCPs on the solid support. Compaction oligonucleotides indeed limited the dimensions of RCPs on the surfaces, although the brightness of the generated RCPs was lower than expected and the number of detected signals was 25-fold lower compared to when 15% PEG 4000 was used (Supplementary Figure S5). The combination of 15% PEG 4000 and compaction oligonucleotides slightly lowered both the number and the average brightness of the generated RCPs compared to 15% PEG 4000 alone. We conclude that the addition of PEG without compaction oligonucleotides is preferable for generating RCPs that can be efficiently recorded.

### Sequential multiplexing

**Figure 4.**
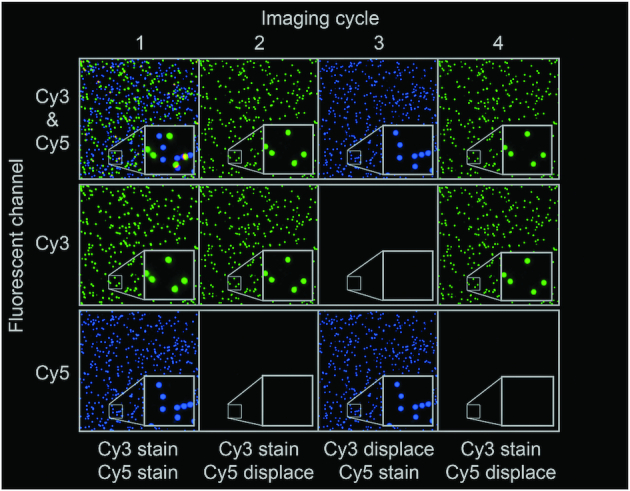
The possibility to perform sequential multiplexing was demonstrated by using two different circular template DNA strands, detected with Cy3- and Cy5-conjugated detection oligonucleotides, respectively. Displacer oligonucleotides were used to remove the staining of previously imaged RCPs via toehold-mediated DNA strand displacement, and in the same reaction new RCPs to be imaged were being stained. Four destaining and staining cycles are shown.

Recording of RCPs on planar surfaces offers an opportunity for digital readout of reaction products in multiplex. We found that using a strand displacement mechanism we could combine the removal of detection oligonucleotides from previously imaged RCPs while simultaneously staining new sets of RCPs, in a process that could be cyclically repeated. To demonstrate the procedure we used two different circular templates, one whose RCPs were specifically recognized by a Cy3-conjugated detection oligonucleotides, while products of the other DNA circle were detected using Cy5 (Figure 4). In a first detection cycle both detection oligonucleotides, each labeled with one of the two fluorophores, were used to stain RCPs of the two specificities. In a second cycle displacement oligonucleotides for the Cy5 labeled probes were combined with Cy3 detection oligonucleotides. In the third cycle the converse combination of reagents was added, i.e. displacement oligonucleotides for the Cy3-labeled probe along with Cy5-labeled detection probes. Finally, in a fourth cycle the same reagents were added as in the second cycle, removing Cy5 probes and staining with Cy3 probes. The displacement oligonucleotides efficiently de-stained the corresponding detection oligonucleotides while simultaneously the other RCP specificity was revealed using the appropriate detection oligonucleotides (Figure 4, two rightmost panels).

### Dynamic range

**Figure 5.**
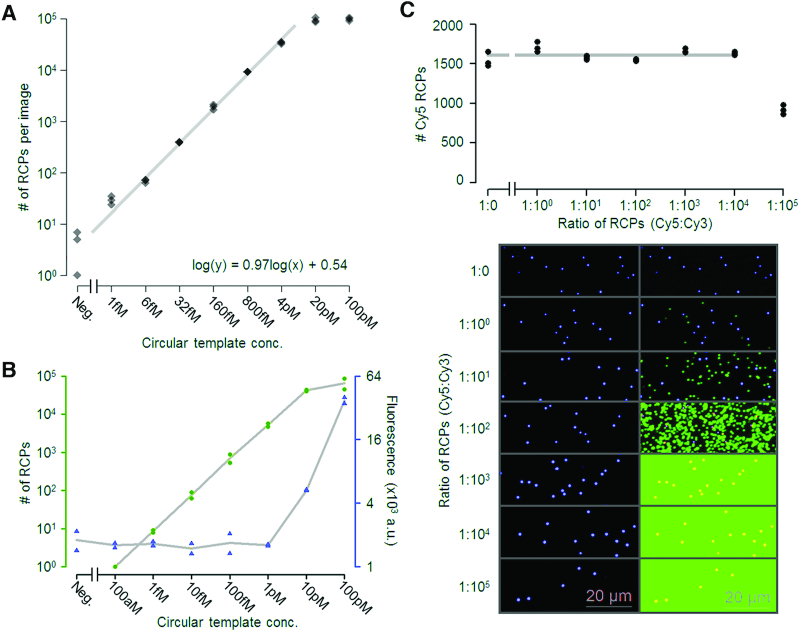
Evaluation of the linearity, dynamic range and saturation of amplification of circular templates on a solid support. (**A**) The dynamic range was evaluated using a five-fold dilution series from 100 pM to 1 fM along with a negative control (immobilization buffer without circular templates). Three images were acquired from each concentration and the recorded numbers of RCPs are displayed in a scatter plot. The grey line represents log-log linear regression calculated from the average numbers of RCPs per image from the five concentrations between 6 fM and 4 pM. The slope of the regression line, at 0.97, is very close to 1, corresponding to perfect absolute quantification. The dynamic range was demonstrated to be around 10 000-fold (1 fM to 10 pM) with excellent linearity. (**B**) The dynamic range of the present method with digital enumeration was compared to the analog measurement of total fluorescence per well using a plate reader, for streptavidin-coated μ-clear 96-well plates. Because of the lower background, several thousand-fold lower amount could be recorded by the digital measurement compared to the analog. (**C**) Detection of RCPs from a particular DNA circle was evaluated as a function of increasing numbers of total DNA circles. 100 fM of circles detected using Cy5-labeled detection probes (blue dots) were mixed at ratios between 1:0 and 1:100 000 with circles detected with Cy3-labeled probes (green dots). Counts of Cy5-labeled RCPs were calculated from six images captured from each ratio. These values were visualized in a scatterplot (top) with Cy5 counts on the y-axis and the added ratios of DNA circles templating RCA displayed on the x-axis. The grey line represents the average value of Cy5-labeled RCPs demonstrating closely similar detection efficiencies in all samples except that where a hundred thousand-fold excess of the DNA circle whose RCPs were detected in green.

With the potential to use RCA on surfaces to analyze biomolecules that may vary over wide concentration ranges, we wished to evaluate over what dynamic ranges measurements can be made. The dynamic range and homogeneity of signals on the solid support were investigated by imaging 5-fold dilutions of circular templates added to the solid support (Figure 5A). Signals representing RCPs were evenly distributed across the surface (the numbers of triplicate determinations are shown as black diamonds). Numbers of signals could be individually resolved, and they increased linearly across a 10 000-fold concentration range (from 1 fM to 10 pM). The results demonstrate the possibility to record the absolute numbers of molecules in a sample based on the calculated log-log linear regression line with a slope of 0.97, close to a theoretical slope of 1. The sensitivity of the present assay is at the single femtomolar level and the area of immobilization can be adjusted according to the reaction volumes.

Computer-assisted counting of RCPs on planar surfaces offers an opportunity to obtain digital measurements of any molecule whose presence in a sample can be represented by the formation of circularized DNA strands. We compared the sensitivity and accuracy between digital and analog measurements of RCPs, by either counting individual RCPs or measuring the integral fluorescence signal across the surface. This comparison was made by coating μ-clear 96-well plate wells with streptavidin, attaching 10-fold dilutions of biotinylated oligonucleotide primers hybridized to circular DNA templates, performing RCA and then recording the results either using digital enumeration via fluorescence microscopy or by measuring total fluorescence in a plate reader (Figure 5B). The results demonstrate that digital enumeration via imaging is far more sensitive by avoiding background signals, and the responses were more proportionate and accurate compared to the analog measurements that only revealed signals above background for the two highest concentrations investigated.

An important aspect that can limit the degree of multiplexing and concentration ranges over which analytes can be measured is inhibition by total surface saturation from the multiple circular template specificities. To evaluate this effect a circular template, recognized by Cy5-conjugated detection oligonucleotides, was analyzed when added together with an increasing number of another circular DNA template capable of hybridizing to the same biotinylated oligonucleotide for surface capture, but detectable using a distinct Cy3-conjugated detection oligonucleotide (Figure 5C). We found that even a 10 000-fold excess of DNA circles whose RCPs were detectable with Cy3 (1 nM) did not affect the number of RCPs detectable with Cy5, added at 100 fM. From 10 pM and higher concentrations of Cy3 signals and could not be individually resolved (green color in lower right images).

### Ultra-sensitive digital measurement of PSA via immuno-RCA

**Figure 6.**
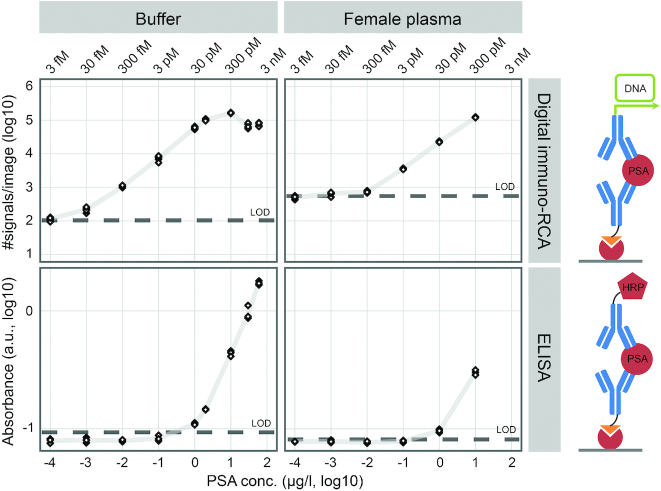
Measurement of dilutions of PSA in buffer and female plasma with digital immuno-RCA and ELISA. The lowest PSA concentration prepared was 100 pg/l and the highest were 60 and 10 μg/l for buffer and female plasma, respectively. Negative controls were used to calculate LOD as three standard deviations above the mean. Six images from each sample were analyzed with digital immuno-RCA and triplicate samples were analyzed using ELISA. More than three orders of magnitude greater sensitivity was achieved when PSA was measured in buffer with digital immuno-RCA (∼100 pg/l) compared to ELISA (∼500 ng/l) (left panels). The difference in sensitivity was not as great for PSA spiked into female plasma, most likely due to low levels of endogenous PSA that could only be recorded using digital immuno-RCA. The same pair of monoclonal anti-PSA antibodies was used for both methods. The capture antibody was conjugated to biotin and the detection antibody was conjugated to DNA or HRP for digital immuno-RCA and ELISA, respectively. The percent values in the upper left panel represent detection efficiencies, i.e. the detected fraction of the added PSA molecules.

To explore the advantages of our digital readout platform we developed a digital immuno-RCA assay targeting PSA, and we compared this to a commercial sandwich ELISA using the same antibody pair (Figure 6). Measurements of PSA titrations in buffer via digital immuno-RCA demonstrated a detection range between 100 pg/l and 10 μg/l, spanning five orders of magnitude (Figure 6, upper left panel). The corresponding values for ELISA were 500 ng/l and 60 μg/l—a more modest 2 orders of magnitude (although, the upper limit was not reached at the highest PSA concentration in the dilution series) (Figure 6, lower left panel). Hence, the sensitivity was more than three orders of magnitude greater when individual detection events were enumerated using digital readout via immuno-RCA compared to analog readout using ELISA. Absolute detection efficiencies (i.e. the fraction of added PSA molecules detected) from the titrations in buffer measured by digital immuno-RCA were on average ∼1% (calculation based on counting RCPs in an image that represented 0.96% of the total sample). Also, titrations of PSA between 100 pg/l and 10 μg/l in pooled female plasma were analyzed (Figure 6, right panels). The unknown endogenous level of PSA in the pooled female plasma sample was estimated to be in the low ng per liter range as measured by digital immuno-RCA but non-detectable as measured by ELISA. The elevated detection level for the non-spiked female plasma compared to buffer when measured with immuno-RCA might partly be due to increased non-specific signals due to the complex matrix. The detection sensitivity for PSA spiked in female plasma was greater using digital immuno-RCA compared to ELISA, but not as striking as for the titration in buffer. This is probably due to endogenous PSA in the female plasma being recorded using the ultra-sensitive digital immuno-RCA assay. The same antibody pair was used for the digital immune-RCA and the ELISA, and the recognition reactions were highly similar. The RCPs generated from individual detection events with immuno-RCA were intense round dots of fluorescence that were easy to enumerate (Supplementary Figure S8). Two different concentrations of detection antibodies were investigated for immuno-RCA (Supplementary Figure S9). Both the background and the true signals decreased approximately 10-fold when the detection antibody concentration was reduced 10-fold, demonstrating very similar detection characteristics at both concentrations.

## DISCUSSION

Building on advances in molecular detection technologies, large sets of biological samples are now being analyzed with respect to vast numbers of molecular features in search of novel biomarkers. The aim is to validate the discovered biomarkers for application in clinical routine, and improved means to read out detection reactions is a high priority. Digital recording of individual target molecules or molecular events in samples ensures the most accurate quantification possible, with digital PCR technologies serving as a gold standard. These technologies rely on individual compartmentalized PCR-based detection reactions as envisaged early on by the inventor of PCR (21). The method uses specialized instrumentation and throughput and dynamic ranges are limited, depending on the number of compartments used. We report herein an alternative approach where individual circular DNA molecules are locally amplified via RCA on planar solid supports and counted by fluorescence microscopy with image analysis. This detection platform presents advantages in the form of reduced reliance on dedicated instrumentation and increased potential for multiplexing and high throughput.

In the present investigation, we demonstrated that 15% PEG ensures excellent RCA efficiency, producing even, rounded RCPs optimal for digital detection when RCA was performed on streptavidin-coated microscope slides. PEG has been used previously for the purpose of molecular crowding in order to allow more efficient enzymatic reactions. In an earlier investigation the ability of PEG to facilitate hybridization of oligonucleotides to be ligated and to support their ligation was seen as a main benefit in RCA of DNA circles immobilized on magnetic beads (12). Our results herein demonstrate the value of including PEG during RCA, both for efficient recognition of the primer-DNA circle complex on the support and for producing evenly shaped RCPs. Our findings pertain to RCA performed on streptavidin-coated microscope slides, while addition of PEG did not improve the numbers or shapes of RCPs generated through *in situ* PLA in cells or tissues or when RCA was performed in solution, according to preliminary results (data not presented). In an earlier study PEG was shown to accelerate the hybridization of oligonucleotides in solution to ones immobilized on gold nanoparticles but no effect was seen on the hybridization of oligonucleotides in solution (22). PEG may interact with surfaces, thereby reducing the tendency of DNA to bind nonspecifically, and thus allowing oligonucleotides to extend from rather than adhere to the surface. This may have the effect of making immobilized DNA more readily available for hybridization and for recognition by enzymes. The rounder shape of RCPs that we observed in a PEG environment may be due to reduced interaction of the RCP with the surface, allowing the products to adopt a uniform random coil shape, observed as point-like fluorescent spots. The shape of the RCPs once formed is maintained also when PEG is removed after RCA.

The focus of our study was to find conditions for efficient enumeration of products of circular DNA strands. Automation could greatly increase throughput, robustness and convenience of the method, especially for high degrees of multiplexing.

RCA performed in solution is sensitive to interfering molecules, e.g. nucleic acids in the sample or added as reagents. If such molecules are not removed via e.g. exonuclease treatment they can act as primers by hybridizing to the replication product, leading to accumulation of both single- and double-stranded amplification products in poorly controlled hyperbranched RCA reactions that are not conducive to digital counting (23). When the circular templates for RCA are attached to a solid support, then RCA conditions can be easily controlled, ensuring optimal digital recording of the data. Nonetheless, it is also possible to perform RCA in solution and to trap products on planar surfaces for example via filtration for digital enumeration of the signals to achieve high accuracy and sensitivity (9,10). Such assays may serve to identify target DNA derived from a fetus with a supernumerary chromosome 21 in the mother's blood for non-invasive prenatal diagnostics (NIPT) (9,10).

A wide range of biomarker classes in various sample types can be analyzed via digital RCA detection, as long as the assays allow the target molecules to be represented by small circular single stranded DNA molecules. Detection of DNA and RNA molecules can result in the formation of amplifiable circular DNA strands via highly specific padlock probes (3,4,24,25,10). Protein assays can yield RCPs via immuno-RCA (5) or through proximity ligation assays that depend on target-binding by pairs of antibodies, thereby ensuring increased specificity of detection and recording of interacting pairs of proteins (8,26). Similarly, linear DNA strands resulting from PCR assays or for example proximity extension protein assays can be converted to DNA circles for readout by digitally counting RCPs. (27,28).

Digital readout offers not only improved accuracy compared to analog measurements but it may also increase sensitivity of detection. We demonstrate herein more than three orders of magnitude greater sensitivity in measuring PSA via digital immuno-RCA assay compared to the corresponding analog ELISA. The procedures for the two assays where kept as similar as possible, e.g. using the same pair of monoclonal antibodies and a similar target recognition and immobilization strategy, in keeping with the recommendations for the commercial assay. The essential difference between the two methods was the readout strategy. Digital immuno-RCA served to enumerate individual detection events through localized fluorescence signals that were clearly distinguishable above background noise. By contrast, ELISA measured global signals as a combination of captured detection reagents and nonspecific background absorbance. The different readout strategies are therefore the main reason for the greatly increased sensitivity achieved with immuno-RCA. In immune-RCA, only detection reagents, whether bound in specific detection reaction or due to cross reactions for off-targets or via nonspecific sticking in the assay wells, can give rise to detectable signals.

Highly sensitive protein assays will likely increase in importance in molecular diagnostics. Such assays can enable detection of trace amounts of leakage biomarkers released into circulation from disease processes anywhere in the body. It is also likely that future disease diagnostics will depend on measurement not of single but of sets of biomarkers, as multiplex investigations can better distinguish between disease states.

PSA is a well-known biomarker in prostate cancer. The ability to measure PSA concentrations at or below ng per liter may enable early detection of relapse for surgically treated prostate cancer patients (29,30) and it can potentially also assist diagnoses of hyperandrogenism in polycystic ovarian syndrome, which is a common cause of reproductive and metabolic dysfunction in women (31). The PSA concentration in the pooled female plasma sample that we analyzed was ∼2 ng/l according to the PSA standards prepared in buffer, as measured with immuno-RCA. Also, one pooled male plasma sample was analyzed demonstrating almost identical values of just below 1 μg/l when measured with immuno-RCA and with ELISA. Both the values for the male and female samples by digital immuno-RCA are in keeping with the literature, stating that circulating PSA, likely originating from breast tissue, is typically present in healthy women at levels <4 ng/l, which is ∼1000-fold lower than normal levels in males (32,33). The detection efficiency of spiked-in PSA was lower in female plasma compared to buffer when analyzed with immuno-RCA. This may be because some of the detection antibodies interact with other plasma proteins. The estimated detection efficiencies (on average around 1%) for detection of PSA in buffer with immuno-RCA are not entirely satisfactory and a potential opportunity to further increase sensitivity by for example increasing the antibody recognition and immobilization time.

In conclusion, the results of our study suggest an alternative means for digital molecular detection reactions besides the gold standard dPCR with benefits such as simplified instrumentation and increased multiplexing capacity and throughput. The readout method presented herein is also suitable to digitally measure a wide range of biomolecule classes, provided that the detection reaction results in DNA circles that may be accurately enumerated after local amplification on solid support via RCA.

## Supplementary Material

gkaa419_Supplemental_Files
