# Supplementary material for: A multiplex platform for digital measurement of circular DNA reaction products
Source: Nucleic Acids Res. 2020 May 29;48(13):e73. doi: 10.1093/nar/gkaa419 (PMC7367203; doi:10.1093/nar/gkaa419)
Supplement: gkaa419_Supplemental_Files [file gkaa419_supplemental_files.zip › Supplementary material - submission version.pdf]

SUPPLEMENTARY MATERIAL

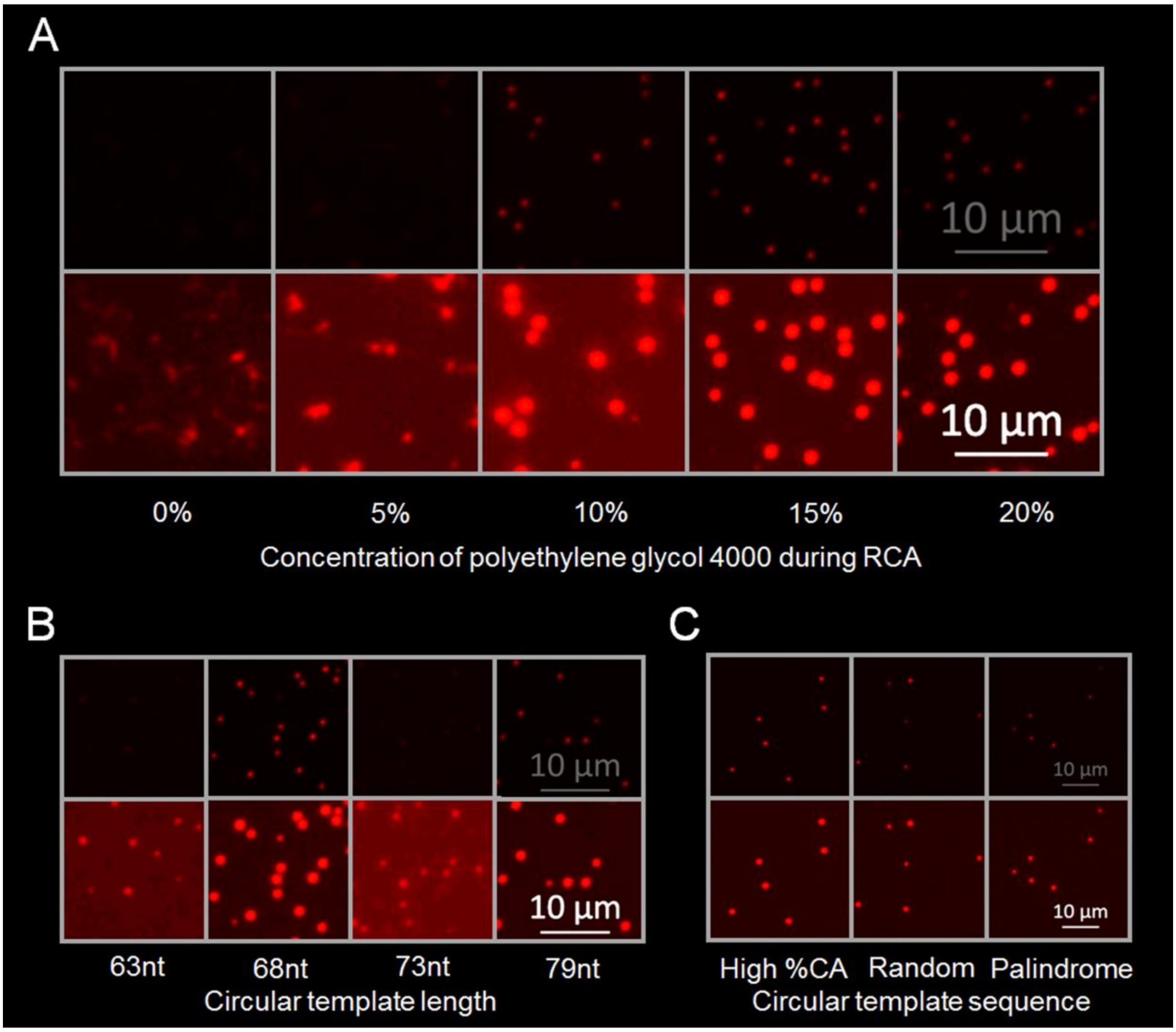

Supplementary Figure S1. Original unaltered microscope images (top panels) are shown together with the corresponding brightened and contrast-enhanced versions (bottom panels). A) RCPs generated in the presence of different concentrations of PEG 4000. B) RCPs generated from circular templates of varying lengths. C) RCPs generated from 68 nucleotide-long circular templates of the indicated sequence compositions.

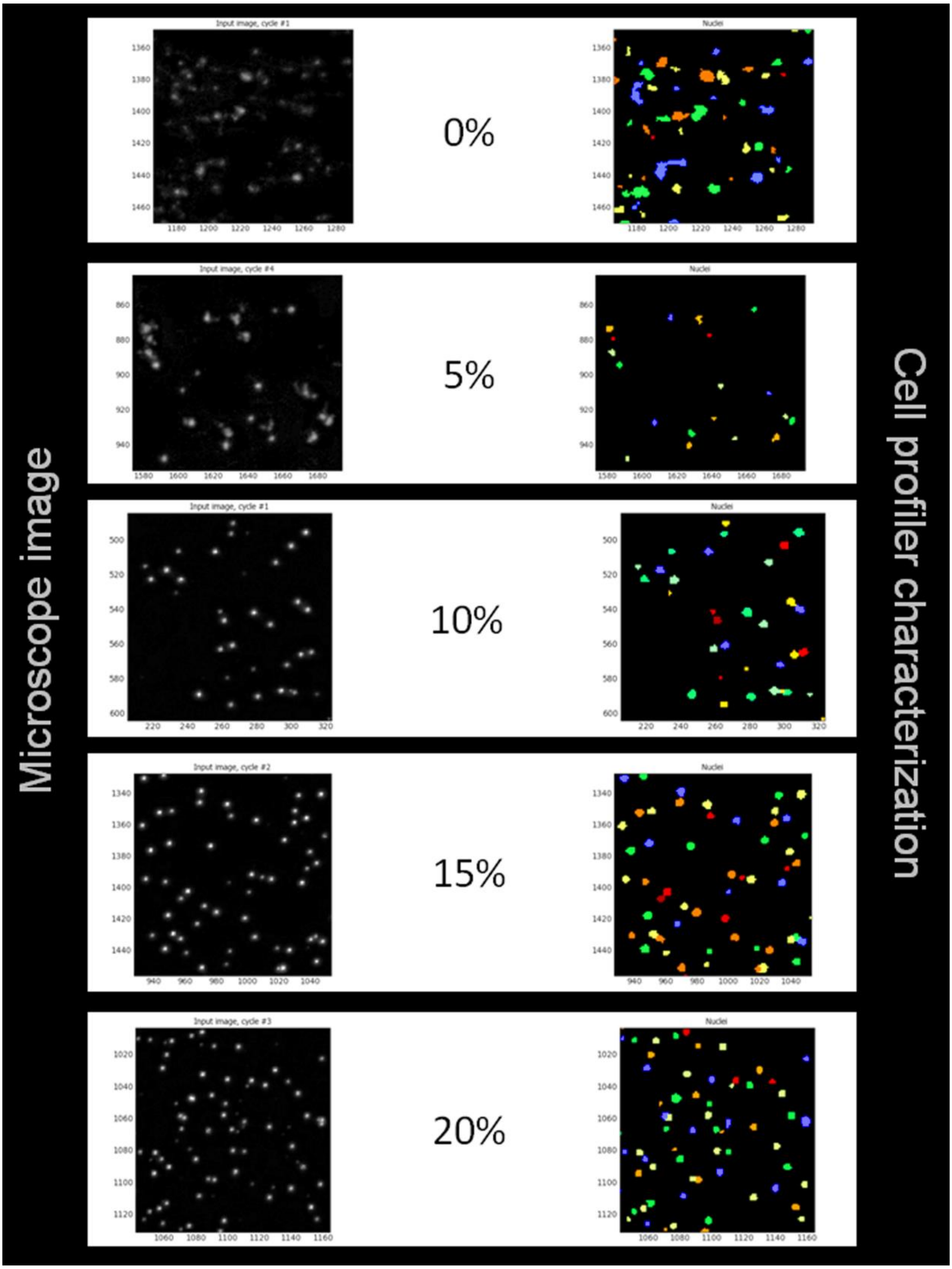

Supplementary Figure S2. The CellProfiler software was used to enumerate and characterize RCPs in the different experiments. A slightly different software pipeline was used to characterize RCPs in samples generated in the presence or absence of PEG, because of the considerable difference in intensity and shape of the RCPs. The left panels represent enhanced images imported into the CellProfiler module IdentifyPrimaryObjects and the right panels are analyzed using CellProfiler software, visualizing the areas of the identified RCPs. The five samples differed in the amount of PEG added; 0, 5, 10, 15 and 20%, respectively.

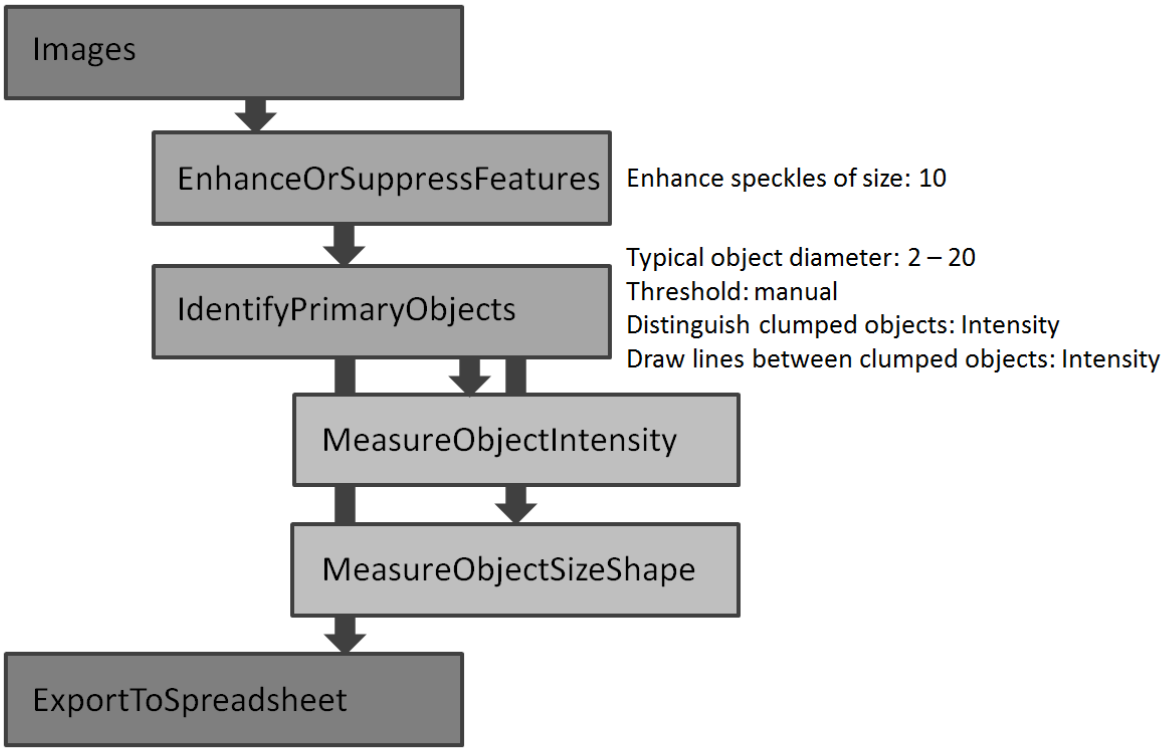

Supplementary Figure S3. Illustration of a typical pipeline used to characterize RCPs with the CellProfiler software. For each image the software enhances RCP-like features, identifies RCPs in the enhanced images, measures the intensity and size of the identified RCPs, and summarizes the data in a spreadsheet. Typical parameter settings used are indicated next to the corresponding modules.

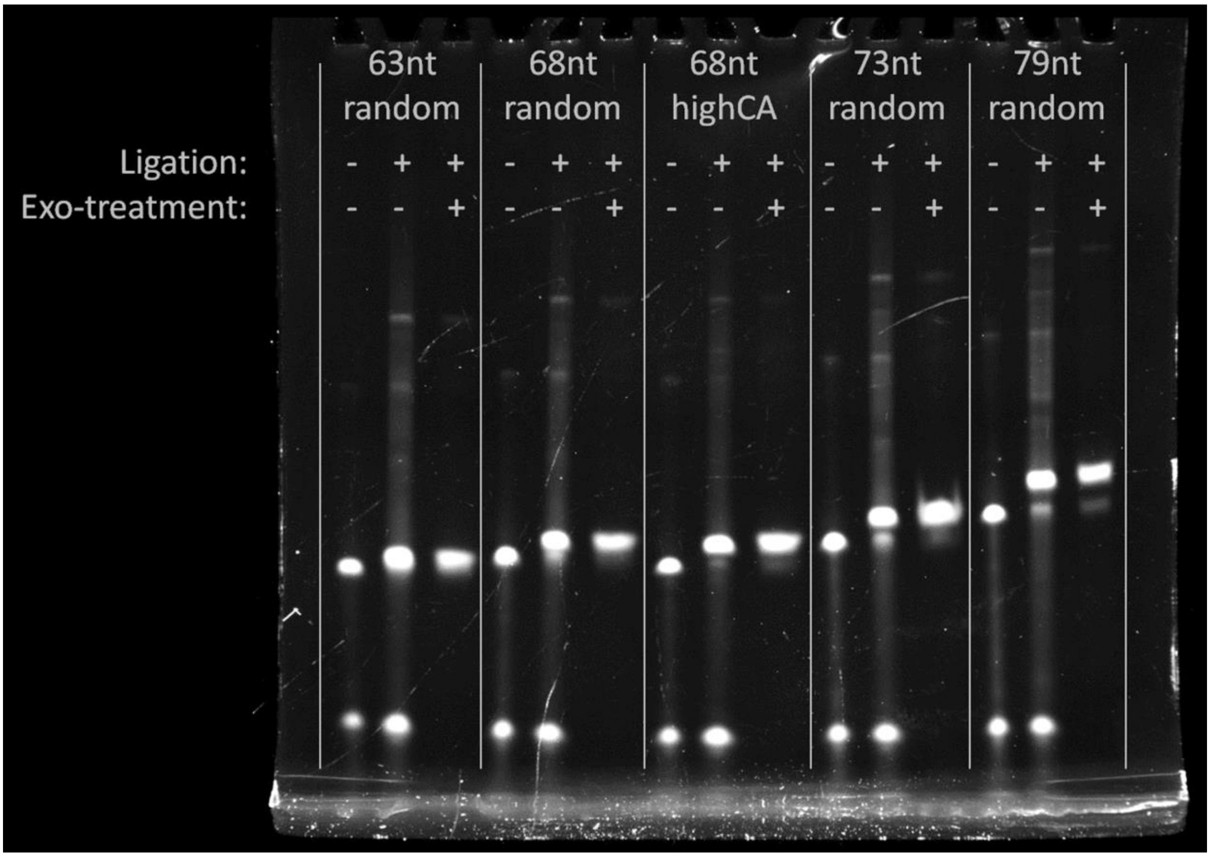

Supplementary Figure S4. The efficiency of circularization by ligation of linear oligonucleotides of the indicated sizes, templated by a 24 nt oligonucleotide, were evaluated by 10% TBE urea polyacrylamide gel electrophoresis. Non-ligated, ligated and exonuclease-treated ligated oligonucleotides were analyzed in separate lanes. Exonuclease treatment was used to degrade all linear DNA molecules. Shorter and linear DNA oligonucleotides migrated faster in the gel compared to longer and circular oligonucleotides, respectively.

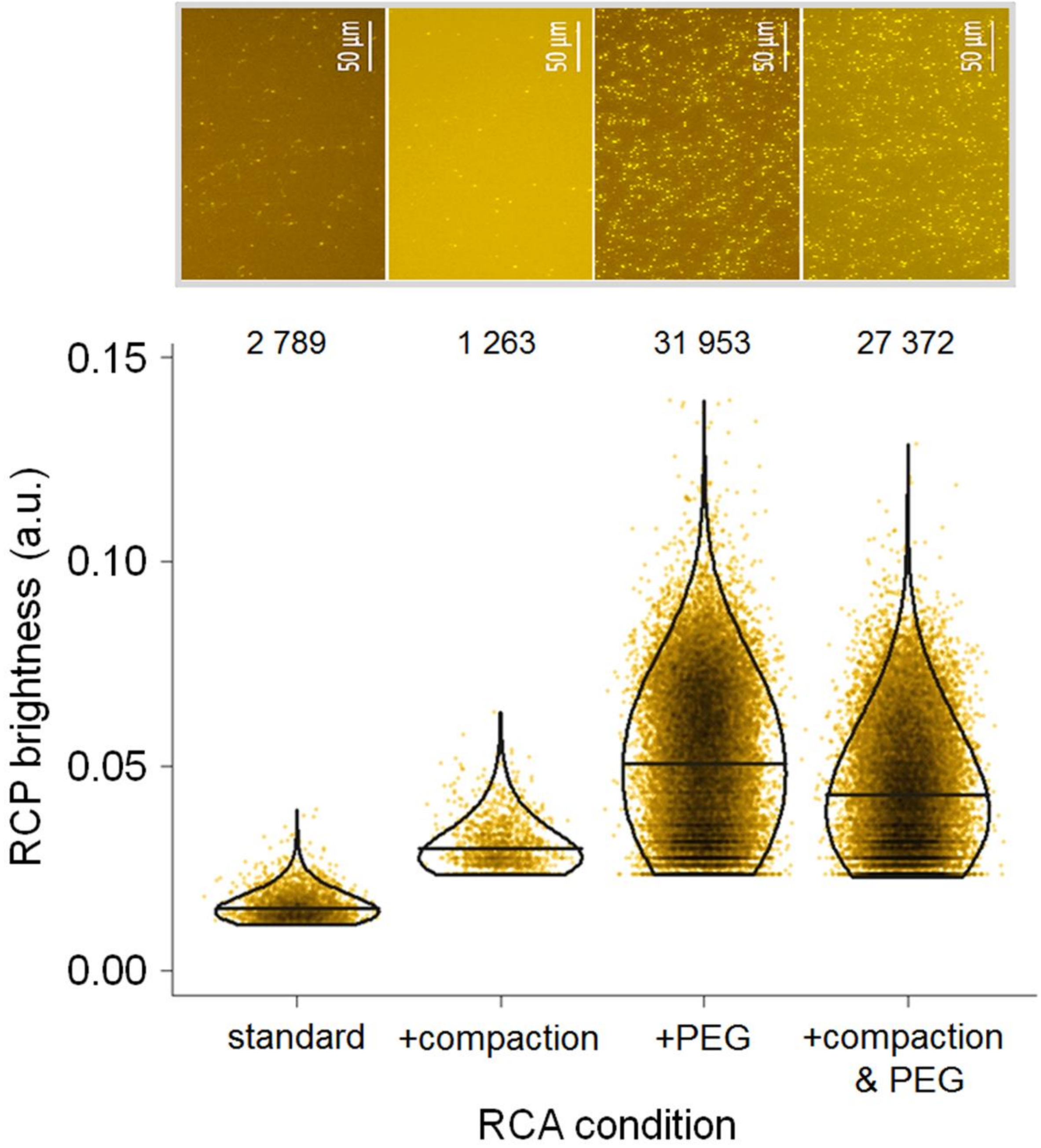

Supplementary Figure S5. The effects on RCP intensity and uniformity by addition of 15% PEG and/or the addition of compaction oligonucleotides were compared. The distribution of brightness by individual RCPs was plotted in the graph and overlaid with violin plots including median brightness. The digitally recorded numbers of RCPs in the different samples are displayed above the corresponding distributions. The images above each distribution are brightened examples of microscope images of the corresponding sample type. PEG allows more circles on the solid support to be detected (greater numbers of signals) and increased the brightness of the generated RCPs (higher median brightness), compared to when no PEG was used, with or without compaction oligonucleotides.

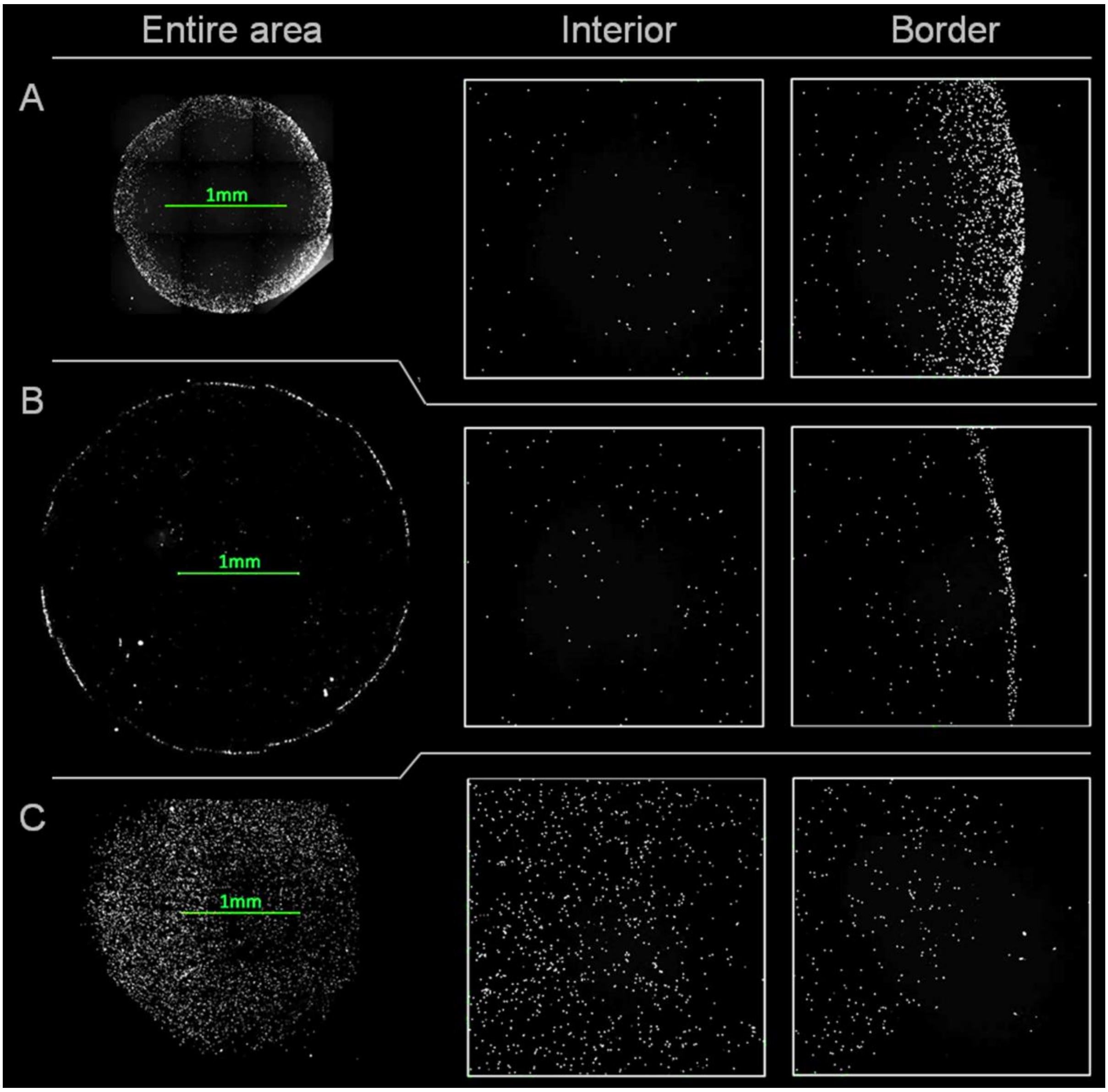

Supplementary Figure S6. The possibility to immobilize circular templates on the solid support via drying was evaluated using three different buffers; A) Phosphate buffered saline (PBS), B) PBS with 0.05% Tween20 (PBST) and C) PBST with 15% PEG 4000. One µl of sample was added to the streptavidin slide and allowed dry for a few minutes in a 45°C incubator. Imaging was performed using a *tiles* function to automatically image the entire area and stitch the captured images together. This was done separately for the three different samples. The same focal plane was used for all images acquired for each sample. Samples suspended in PBS and PBST extended over the smallest and largest entire sample area, respectively and the distribution suffered from "coffee stain effects" (enrichment of stained products at the periphery). PBST with 15% PEG generated the sample area with the most evenly spread RCPs and was considered the best option for immobilization via drying. Although immobilization by drying was fast, the efficiency of immobilization and detection by drying was only around 1% for PBST with 15% PEG.

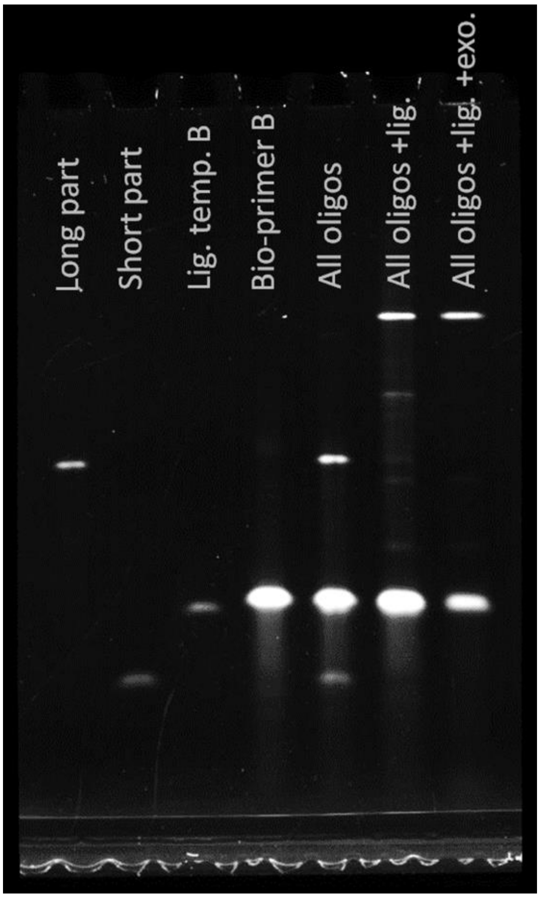

Supplementary Figure S7. Success rate of ligation for system B (Table 1), evaluated by denaturing PAGE. The different oligonucleotides to be ligated (lane 1-4), the non-ligated combination of oligonucleotides (lane 5), the ligation product (lane 6) and exonuclease treated ligation product (lane 7) were run on a 10% TBE urea gel.

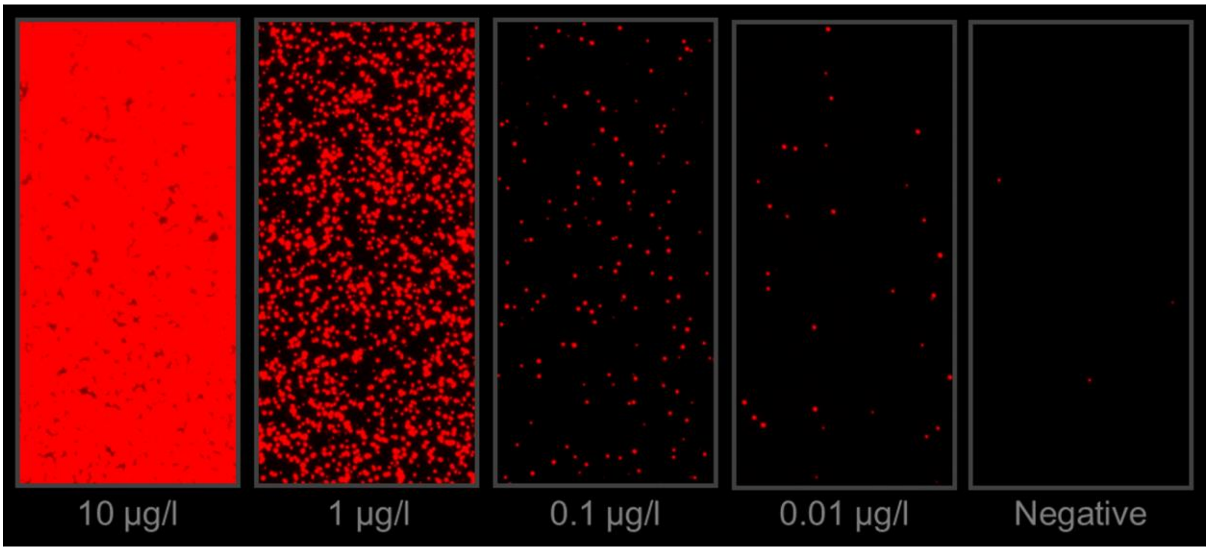

Supplementary Figure S8. Intense and round distinct signals were generated from individual immuno-RCA detection events. The images represent sections of Cy3-channel microscope images with enhanced brightness for the purpose of visualization. The images are examples of four different PSA concentrations in buffer together with the blank buffer used as a negative control.

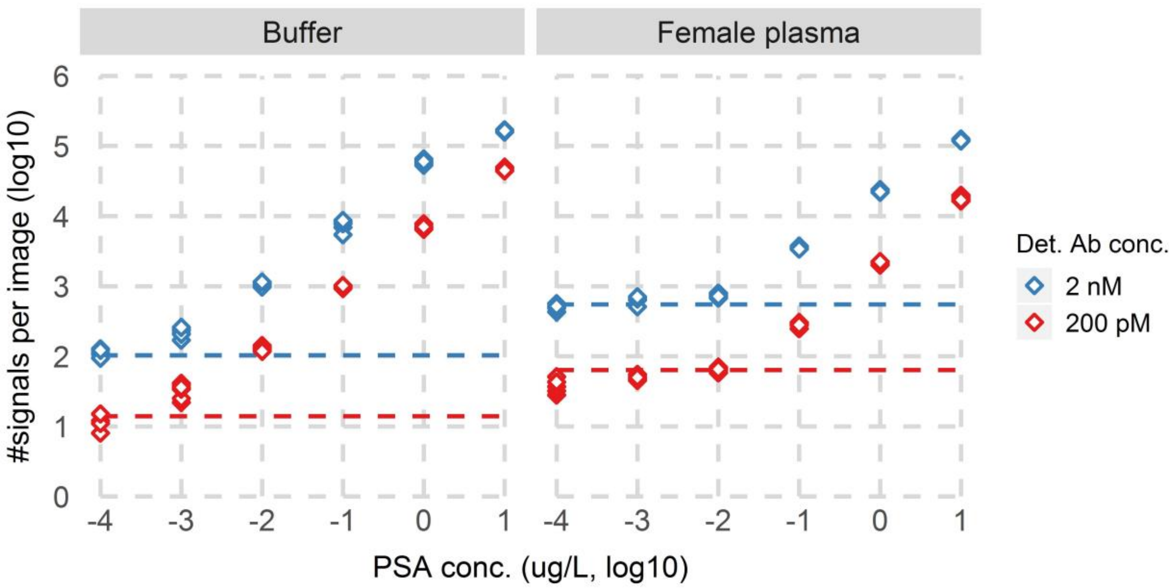

Supplementary Figure S9. Two different concentrations of detection antibody (2 nM and 200 pM) were investigated for immuno-RCA detection of PSA. Both the LODs (dashed lines) and the levels of signals were approximately 10-fold higher for 2 nM detection antibody compared to 200 pM. The higher detection antibody concentration resulted in lower variability of counts per image at higher counts.
